# Exceptionally large entropy contributions enable the high rates of GTP hydrolysis on the ribosome

**DOI:** 10.1038/srep15817

**Published:** 2015-10-26

**Authors:** Johan Åqvist, Shina C.L. Kamerlin

**Affiliations:** 1 Dept. of Cell & Molecular Biology, Uppsala University, Biomedical Center, Box 596, SE-751 24 Uppsala, Sweden

## Abstract

Protein synthesis on the ribosome involves hydrolysis of GTP in several key steps of the mRNA translation cycle. These steps are catalyzed by the translational GTPases of which elongation factor Tu (EF-Tu) is the fastest GTPase known. Here, we use extensive computer simulations to explore the origin of its remarkably high catalytic rate on the ribosome and show that it is made possible by a very large positive activation entropy. This entropy term (*T***Δ*****S***^‡^) amounts to more than 7 kcal/mol at 25 °C. It is further found to be characteristic of the reaction mechanism utilized by the translational, but not other, GTPases and it enables these enzymes to attain hydrolysis rates exceeding 500 s^−1^. This entropy driven mechanism likely reflects the very high selection pressure on the speed of protein synthesis, which drives the rate of each individual GTPase towards maximal turnover rate of the whole translation cycle.

The key chemical step involved in tRNA selection on the bacterial ribosome is the hydrolysis of GTP by elongation factor Tu (EF-Tu)^1^, which commits cognate aminoacyl-tRNAs to accommodate into the peptidyl transferase center so that the subsequent peptide bond formation can take place^2^. That is, upon delivery of aminoacyl-tRNA in ternary complex with EF-Tu and GTP to the ribosome, codon-anticodon recognition is established and this event activates EF-Tu for rapid hydrolysis of GTP, after which EF-Tu•GDP dissociates so that tRNA accommodation can occur. That there is something special about GTP hydrolysis on the ribosome is suggested by the fact that EF-Tu is the fastest known GTPase, with reaction rates exceeding 500 s^−1^ 3,4,5,6. Likewise, the GTP hydrolysis step catalyzed by elongation factor G is very fast with a reported rate constant of 250 s^−1^ at 37 °C^7^. In comparison, the moderately fast Ras family of GTPases is only able to reach catalytic rates^8^,^9^ on the order of 20 s^–1^. This most likely reflects the extraordinary selection pressure exerted on the speed of protein synthesis, due to the size and abundance of ribosomes, which directly determines the growth rate of bacteria. The translation system thus represents by far the largest mass fraction of any enzymatic system in the cell. Hence, if the translational GTPases would be limited by rates as low as 20 s^−1^, this would severely slow down protein synthesis and be incompatible with the required overall translation cycle rates *in vivo*^4^,^6^ of at least 20 s^–1^. Another contributing factor may be that high GTP hydrolysis rates are also required to compensate for competition between cognate and near-cognate ternary complexes^10^. A central question is thus what it is that makes the translational GTPases achieve their unparalleled high GTP hydrolysis rates.

To answer this question, we have recently investigated the detailed mechanism of activation and GTP hydrolysis by EF-Tu, based on crystallographic data for the activated GTPase complex bound to the 70S bacterial ribosome^11^,^12^, using molecular dynamics (MD) simulations in combination with the empirical valence bond (EVB) method for describing the chemical reaction^13^,^14^. These simulations allowed identification of the operational reaction mechanism (among several possible ones) and also reproduced the effects of several key EF-Tu mutations. In order to explore the thermodynamic and structural origin of the enormous catalytic effect of EF-Tu on GTP hydrolysis it is very useful to try to decompose the activation free energy into its enthalpic and entropic contributions. Here we employ our recently developed approach^15^,^16^ of calculating Arrhenius plots from multiple simulations of reaction free energy profiles to attack this problem. The simulations reveal an exceptionally favourable entropy contribution to the catalytic rate which is found to be associated with the specific reaction mechanism used by EF-Tu.

## Results

### Calculations of Arrhenius plots for GTP hydrolysis

**Figure 1:**
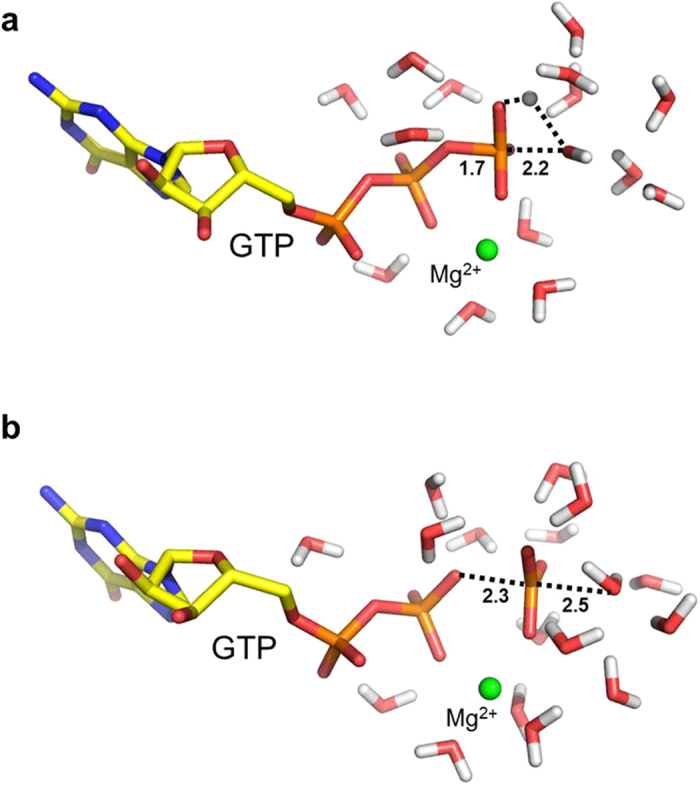
EVB models for uncatalyzed GTP hydrolysis in water. Representative snapshots of the transition state ensemble from MD simulations of the (**a**) associative and (**b**) dissociative variants of the uncatalyzed solution reaction^14^,^21^. Only water molecules near the β- and γ-phosphates are shown for clarity. The Mg^2+^ ion is depicted as a green sphere.

**Figure 2:**
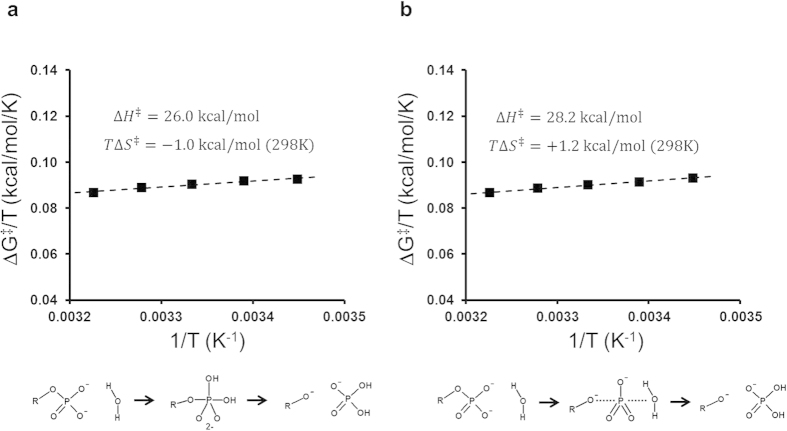
Calculated Arrhenius plots for the uncatalyzed hydrolysis of Mg^2+^•GTP^4-^ in water. The two limiting mechanistic cases corresponding to associative (**a**) and dissociative (**b**) reaction pathways^19^,^20^,^21^ are considered. For each mechanism an EVB model was calibrated to reproduce a free energy barrier of 27 kcal/mol (at 298 K and a 55 M standard state of water)^14^. Five independent free energy profiles were calculated for each mechanism at each temperature. The s.e.m. for the calculated free energy barriers at a given temperature ranges between 0.03 and 0.19 kcal/mol.

We carried out computer simulations of the temperature dependence of both the uncatalyzed GTP hydrolysis reaction in solution and the EF-Tu catalyzed reaction on the ribosome. These simulations employed the empirical valence bond (EVB) method^17^,^18^, as described earlier^14^, and a large number of reaction free energy profiles were calculated at different temperatures. Analysis of the computed results, in terms of Arrhenius plots relating the activation free energy to temperature (Δ*G*^‡^/*T* versus 1/*T*), allows the activation enthalpy (Δ*H*^‡^) and entropy (Δ*S*^‡^) to be extracted with sufficiently high precision^16^. The mechanism of uncatalyzed hydrolysis of GTP in aqueous solution has been debated^19^,^20^ and was modelled here as proceeding through either a dissociative or an associative transition state (TS) (Fig. 1)^14^,^21^. The computational Arrhenius plots for both variants of the uncatalyzed mechanism (Fig. 2) yield activation entropies close to zero, in excellent agreement with experiment^22^,^23^. The values of *T*Δ*S*^‡^ (at 298 K) for hydrolysis of Mg^2+^•GTP^4−^ are predicted to be +1.2 and −1.0 kcal/mol for the dissociative and associative mechanisms, respectively, which can be compared to the experimentally derived value^22^ of −0.8 kcal/mol (the corresponding value for ATP hydrolysis has been reported as −1.9 kcal/mol)^23^.

**Figure 3:**
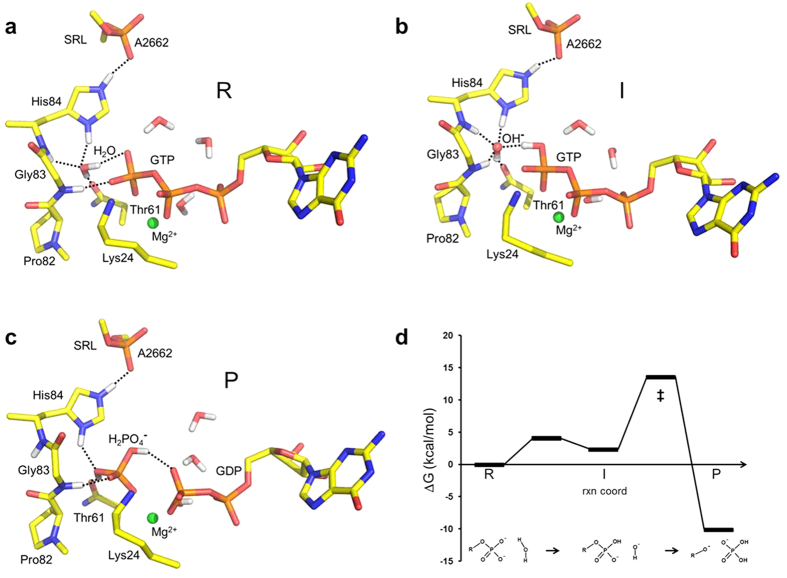
Stabilization of the nucleophile in the active site of EF-Tu. Proton transfer from a catalytic water molecule^11^,^12^,^13^,^14^ to the γ-phosphate of GTP is strongly favoured on the ribosome. This is illustrated by representative MD snapshots at (**a**) the reactant (R) state, (**b**) the intermediate (I) resulting from proton transfer and (**c**) the product state with inorganic phosphate and GDP bound (key hydrogen bonds are shown with dashed lines). The corresponding average free energy profile obtained form 15 independent simulations at 300 K is also shown (**d**). Key amino acid residues of EF-Tu are indicated together with the A2662 phosphate group of the sarcin-ricin loop (SRL) from the large ribosomal subunit. The Mg^2+^ ion (green sphere) bridging between the β- and γ-phosphate groups and nearby water molecules are also shown.

For the EF-Tu catalyzed process on the ribosome, in contrast, we have recently shown that the only reaction pathway compatible with experimental kinetics^3^,^4^,^5^,^6^,^24^ involves protonation of GTP and attack by hydroxide ion on the γ-phosphate^13^,^14^. This mechanism was further found to be facilitated by the conformation of the universally conserved PGH (Pro-Gly-His) motif at the catalytic center of the translational GTPases (Fig. 3, Supplementary Fig. 1). Here, both the backbone orientation of the motif and the positioning of the critical histidine sidechain (His84 in *E. coli* EF-Tu) are essential for catalysis^13^,^14^,^24^ and the main catalytic effect is a huge stabilization of the hydroxide ion state. Recent atomic mutagenesis experiments^25^, that removed the negative charge on the sarcin ricin loop phosphate group which interacts with the protonated histidine, provide additional support for this mechanism.

**Figure 4:**
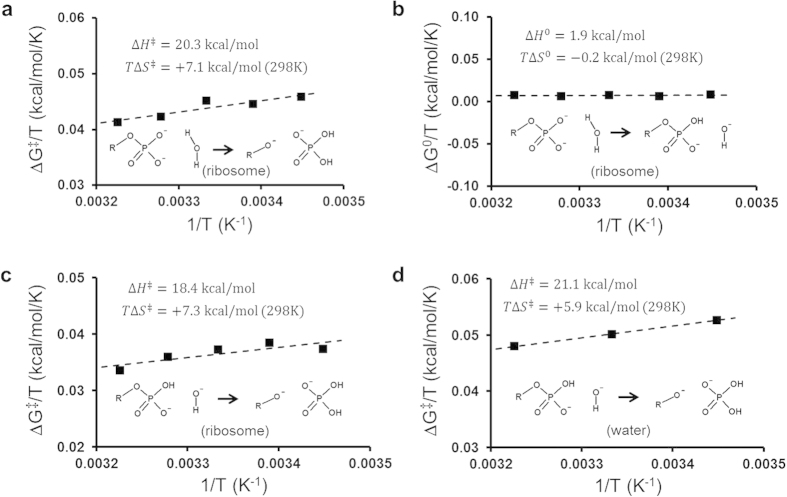
Simulated Arrhenius and van’t Hoff plots for GTP hydrolysis by EF-Tu on the ribosome. (**a**) Calculated Arrhenius plot, relating the activation free energy (Δ*G*^‡^) to inverse temperature, for the overall GTP hydrolysis reaction on the ribosome and its breakdown into proton transfer (**b**) and nucleophilic attack steps (**c**). (**b**) van’t Hoff plot of the temperature dependence of the reaction free energy (Δ*G*^0^) for proton transfer from the catalytic water molecule to the γ-phosphate of GTP. (**c,d**) Calculated Arrhenius plots for attack of OH^−^ on the protonated γ-phosphate on the ribosome and in water, respectively (the standard state for the OH^−^ attack in water is 55 M in order to be directly comparable to ribosome result). Each temperature point in panels (**b–d**) represents the average of 15 independent simulations. The s.e.m. for the calculated free energies at a given temperature ranges between 0.26 and 0.39 kcal/mol for the ribosome plots and between 0.09 and 0.16 kcal/mol for the OH^−^ attack in water.

Our calculated Arrhenius plots for EF-Tu catalyzed GTP hydrolysis (Fig. 4a–c) display good fits to straight lines in the temperature interval 290–310 K. The cost of proton transfer from the water nucleophile to the γ-phosphate group of GTP is very low (Fig. 4b) and mainly enthalpic, with only a small entropy contribution (*T*Δ*S*^0^ = −0.2 kcal/mol at 298 K). This is also in line with the small entropy effect found experimentally for protonation of both inorganic phosphate dianion and Mg^2+^•GTP^4−^ by water in aqueous solution, which can be calculated from thermodynamic data^26^,^27^ to be *T*Δ*S*^0^ = +0.9 and −1.5 kcal/mol at 298 K, respectively. In contrast, the subsequent OH^−^ attack on the protonated GTP molecule on the ribosome is predicted to occur with a relatively high enthalpy penalty (Δ*H*^‡^ = 18.4 kcal/mol) that is counterbalanced by an extraordinarily favourable activation entropy corresponding to *T*Δ*S*^‡^ = +7.3 kcal/mol at 298 K (Fig. 4c). Interestingly, the origin of this large positive activation entropy (25 e.u.) is clearly the delocalization of the hydroxide negative charge over the entire γ-phosphate in the TS, compared to its more localized character in the reactant state for the attack. That is, it is the surrounding active site environment whose entropy increases when it does not need to strongly “solvate” the hydroxide ion anymore (Fig. 3b). This conclusion can be further corroborated by examining the same uncatalyzed OH^−^ attack on protonated Mg^2+^•GTP^3−^ in aqueous solution. The corresponding Arrhenius plot is shown in Fig. 4d, where it can be seen that the activation enthalpy is higher than in EF-Tu as expected, but that *T*Δ*S*^‡^ is still large and positive (+5.9 kcal/mol at 298 K), and in this case it is evidently the increased configurational entropy of the solvent in the TS that causes the effect. This high positive *T*Δ*S*^‡^ is also in good agreement with that reported for hydrolysis of methylphosphate under highly alkaline conditions, where OH^−^ attack on the monoanion presumably dominates the reaction^28^.

Hence, our simulations predict that GTP hydrolysis on the ribosome proceeds with an overall activation entropy corresponding to *T*Δ*S*^‡^ = +7.1 kcal/mol at 298 K (Fig. 3a), which is distinctly more favourable than that of the peptidyl transfer reaction^4^,^29^. In fact, the entropy contribution to the GTPase rate appears to be larger than observed for any other enzyme at physiological temperatures^30^,^31^. Furthermore, our prediction is in strikingly good agreement with the temperature dependence of *k*_cat_ for GTP hydrolysis reported by Ehrenberg and coworkers^4^. From their rates at 283, 288 and 293 K an activation entropy of *T*Δ*S*^‡^ = +7 kcal/mol can be estimated and the same result is obtained from the data in ref. 6. Here, it should be noted that fluorescence measurements using mant-dGTP and proflavin-labelled tRNA show a rapid fluorescence increase whose time constant coincides with that of GTP hydrolysis^3^,^5^,^32^. This has been interpreted such that a conformational change associated with GTPase activation may actually be rate-limiting for the hydrolysis reaction^3^,^5^,^32^. Although the exact nature of this activation step is not entirely clear, it is evident that GTP hydrolysis is initiated by conformational change of EF-Tu. This conformational change follows after codon recognition and involves the ternary complex making contact with the sarcin-ricin loop of the large ribosomal subunit, where His84 plays a critical role^3^,^5^,^11^,^13^,^24^.

### Interpretations of favourable entropy effects

**Figure 5:**
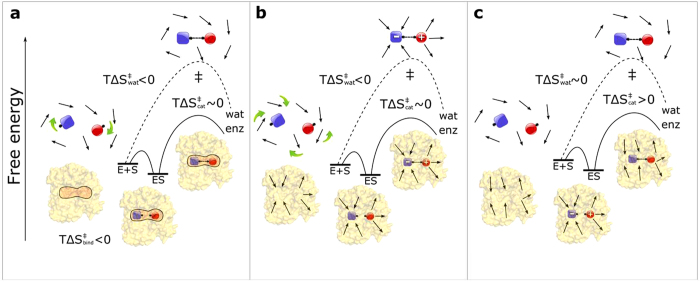
Different possible origins of favourable activation entropies in enzymes. An uncatalyzed reaction in water (wat) is compared to that catalyzed by an enzyme (enz), where the latter has a more positive value of the activation entropy 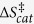 for the chemical step. If the corresponding activation entropy in water, 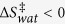, this term is considered to be dominated by reorientation of either (**a**) the substrates or (**b**) the solvent (dipolar groups are represented by arrows). In the former case, the entropy loss can be realized already upon substrate binding in a reactive orientation^33^ and the activation free energy barrier can thus be climbed without further entropy penalty (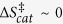). When solvent reorientation is dominant (**b**), which is typical for reactions involving transition states that are more polar than the reactants, binding to an electrostatically preorganized active site^17^ can reduce 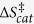 by eliminating the entropy penalty of solvent reorientation. The third case (**c**) depicts a situation with similarly small charge separation in the reactant and transition states for the uncatalyzed reaction that has no significant entropy penalty (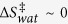). The enzyme can nevertheless increase 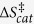 by stabilizing a more polar reactant state (ES) than the TS. That is, if charges in the TS are more delocalized than in ES, then the orientational entropy of enzyme active site groups may increase as the barrier is climbed. This is represented by the protein dipoles being more organized in the ES complex than in the TS.

The role of entropy in enzyme catalysis and its connection to reaction rate enhancement has been widely debated for several decades and still remains somewhat enigmatic. In cases where the catalytic effect of an enzyme can be ascribed to a change in activation entropy, i.e., ΔΔ*S*^‡^ > 0 relative to the uncatalyzed reaction, this has often been interpreted in terms of the *substrate* entropy change being more favourable in the enzyme (Fig. 5a). That is, it is assumed that part of the substrate binding free energy is spent on restricting the substrate motions and correctly aligning it for reaction, which implies a negative binding entropy. This, in turn, would enable the substrate to climb the activation barrier with a smaller entropy loss than in solution, since the entropic penalty for the reaction has already been paid upon binding. Accordingly, the catalytic rate constant (*k*_*cat*_) would be characterized by a more positive value of *T*Δ*S*^‡^ than the corresponding uncatalyzed rate. This is one variant of Jencks’ famous “Circe effect”^33^ and it has, for example, been invoked to explain the catalytic efficiency of peptide bond formation on the ribosome^34^. However, there are alternative and distinctly different interpretations of a favourable enzyme activation entropy change (ΔΔ*S*^‡^ >0 ), which instead focus on the entropy of the surrounding *protein* and *solvent* (Fig. 5b,c). Note that the concentration effect of bringing two reacting groups from a 1 M standard state in water into van der Waals contact (*T*Δ*S* ~ −2 kcal/mol)^17^,^18^ is implicit in all cases in Fig. 5.

Indeed, Wolfenden, Rodnina and coworkers^29^ showed that ribosome catalyzed peptide bond formation with the small puromycin substrate proceeds without any entropy loss (*T*Δ*S*^‡^ ~ +1 kcal/mol at 25 °C), while the uncatalyzed reaction is associated with a very large entropy penalty (*T*Δ*S*^‡^ = −13 kcal/mol). However, the observation that *k*_*cat*_/*K*_*M*_ displays approximately the same *T*Δ*S*^‡^ contribution as *k*_*cat*_ on the ribosome suggests that this entropy effect does not originate from substrate alignment and proximity in the Michaelis complex (Fig. 5a). Ehrenberg and coworkers^4^ further showed that ribosomal peptidyl transfer (*k*_pep_) with full-length tRNAs also proceeds with a small and positive activation entropy and that the overall *K*_M_ for dipeptide formation is not either associated with any significant apparent entropy contribution. Instead, the large reduction of the activation entropy cost on the ribosome was ascribed to a preorganized (Fig. 5b) hydrogen bond network in the peptidyl transferase center, involving a number of conserved water molecules in addition to polar ribosomal groups^35^,^36^. This conclusion was based on MD simulations that qualitatively reproduced the observed entropy effect and predicted the existence of the water mediated network^35^, which was later confirmed by high resolution X-ray structures^37^.

The present computer simulations show that the large positive entropy of activation predicted for GTP hydrolysis on the ribosome is not either due to substrate strain or freezing of motions in the reactant state^33^. Rather, it is simply due to a change of mechanism (Fig. 5c) compared to the Mg^2+^•GTP^4-^ hydrolysis reaction in solution. That is, both from our calculations and from experiments^22^,^23^ one finds that the uncatalyzed process (Fig. 2) proceeds with a near-zero activation entropy, while the OH^−^ attack mechanism utilized on the ribosome already in solution would be associated with a large positive *T*Δ*S*^‡^ term^28^ (Fig. 4c). For comparison, the Ras-RasGAP GTPase is generally considered to operate through the type of mechanisms^14^,^15^ shown in Fig. 2, with no strongly charge-separated intermediate state involved. Although that enzyme was also reported to have a large positive activation entropy at very low temperatures (−18 to −8 °C)^38^ its reported rate^8^,^9^ at 25 °C indicates a much smaller contribution. It thus appears that it is precisely the OH^−^ mechanism found in EF-Tu^13^,^14^ that allows the GTP hydrolysis rate to be pushed to above 500 s^−1^. That is, the speed of this mechanism can be largely accelerated by a strong stabilization of the transient reaction intermediate with OH^−^ and a protonated γ-phosphate. Since the OH^−^ attack is then intrinsically relatively fast (estimated to be ~1 M^−1^ s^−1^ in solution^14^) only a few kcal/mol of additional TS stabilization is necessary for reaching very high rates. Hence, it is possible that the universally conserved catalytic PGH motif of the translational GTPases (Fig. 3) may have specifically evolved in order to enable the hydroxide mechanism, thereby making these enzymes extraordinarily fast.

## Discussion

The present computer simulations predict an unusually large and favourable entropy contribution to the activation free energy for GTP hydrolysis by EF-Tu on the ribosome, which enables the extraordinarily high catalytic rate. It is further found that this entropy effect is caused by the specific reaction mechanism utilized by EF-Tu, where the positively charged His84 sidechain is the key determinant of the reaction pathway^14^. The histidine residue is part of the PGH motif (within the so-called G3 motif) which is universally conserved among the translational GTPases and is unique to the translation factor superfamily^39^,^40^. GTP hydrolysis by EF-Tu on the ribosome shows no pH-dependence^5^,^24^, consistent with computational predictions^13^,^41^,^42^ that the p*K*_a_ of His84 is upshifted so that the sidechain is positively charged at neutral pH. Mutation of His84 in EF-Tu has also been shown to severely impair the GTPase activity^5^,^24^. Moreover, experiments on EF-G that replaced the negatively charged phosphate group (A2662) of the sarcin-ricin loop, which interacts with the histidine sidechain, by a neutral methylphosphonate showed significantly reduced activity. Taken together, these results indicate that the histidine residue is central to the catalytic mechanism and that it needs to be protonated (positively charged) for efficient catalysis. This is consistent with its role in stabilizing negative charge on the nucleophile and our earlier calculations on EF-Tu also predicted significant increases in the activation barrier if His84 was either mutated or deprotonated^13^,^14^. Mutation of the nearby Asp21 residue to alanine, on the other hand, was predicted to stabilize the double negative charge on the γ-phosphate of GTP, thereby disfavouring proton transfer from the catalytic water molecule to the phosphate group^14^. The D21A mutation could also potentially downshift the p*K*_a_ of His84 so that the histidine becomes neutral, in which case a concerted water attack becomes the favoured mechanism^14^. Interestingly, both of these scenarios yielded a predicted activation barrier increase^14^ of 4–5 kcal/mol, in agreement with the experimental observations^24^.

The question of what limits the rate of GTP hydrolysis by EF-Tu on the ribosome remains to be investigated in more detail. The present calculations have only addressed the chemical step and the fact that our predicted activation entropy term (*T*Δ*S*^‡^ = +7.1 kcal/mol at 298 K) coincides perfectly with that derived from the observed temperature dependence of GTP hydrolysis^4^,^6^ suggests that the experimental kinetics may actually probe the chemical step. On the other hand, the fluorescence measurements of Rodnina and coworkers^3^,^5^,^32^ report on conformational changes involving the fluorophores and their environment, on the same time scale as the overall GTP hydrolysis process. These conformational changes have been assigned to the GTPase activation step, but whether this process is more diffusive-like or associated with a single dominating free energy barrier remains unclear. In order to further dissect the overall GTP hydrolysis process it would be of considerable interest to determine the temperature dependence also of the rate the fluorescence increase associated with GTPase activation. With regard to the overall rate of GTP hydrolysis it seems quite possible that the activation process and the chemistry take place on similar time scales, with both contributing to the observed catalytic rate.

## Methods

### MD simulations

Molecular dynamics simulations were performed as reported earlier^13^,^14^ with the program Q^43^, utilizing the OPLS-AA force field (Macromodel 9.1, Schrödinger LCC, New York)^44^. In brief, spherical simulations systems (40 Å in diameter) centered on the γ phosphorous atom of GTP were used after solvation by a TIP3P water droplet of the same size. In the ribosome simulations, protein and RNA atoms outside the simulation sphere were tightly restrained to their initial coordinates and excluded from non-bonded interactions. Water molecules close to the sphere boundary were subjected to radial and polarization restraints according to the SCAAS model^43^,^45^. Initial coordinates, based on the crystal structure of the ribosome bound EF-Tu ternary complex^11^ (PDB codes: 2XQD, 2XQE), were taken from our previously reported equilibrated simulation system including solvent and counter ions^13^,^14^. MD simulations were carried out with a 1fs time step and a 10 Å cutoff was employed for direct non-bonded interactions, with electrostatic interactions beyond the cutoff treated by the local reaction field multipole expansion method^46^. No cutoff was applied to non-bonded interactions involving the central part of the system that is treated by the EVB model (*i.e.* the triphosphate moiety of GTP and the hydrolytic water molecule). The reference simulations of different Mg^2+^•GTP^4−^ hydrolysis mechanisms in pure water were also carried out as reported previously^13^,^14^, with solvent spheres of the same size (40 Å in diameter).

### Computational Arrhenius plots and EVB models

Calculations of reaction free energy profiles utilized the free energy perturbation (FEP) umbrella sampling method to drive the system between the different EVB states^13^,^14^. Each such simulation consisted of an MD equilibration phase with step-wise heating from 1 K to 300 K while gradually releasing restraints on heavy solute atoms, followed by 800 ps of unrestrained equilibration at the given temperature. The calculation of each free energy profile then involved 1.1 ns of MD sampling, which comprised 21 intermediate FEP windows. For both the proton transfer step and the subsequent nucleophilic attack on the protonated GTP molecule on the ribosome 15 independent free energy profiles were calculated at each of the five temperatures (290 K, 295 K, 300 K, 305 K and 310 K). These replicate simulations were assigned different randomized initial velocities during equilibration, according to the Maxwell distribution. Averaging of these free energy calculations for each temperature yielded reaction and activation free energies with a standard error of the mean (s.e.m.) of about 0.3 kcal/mol, which is a sufficiently good precision for obtaining reliable van’t Hoff and Arrhenius plots^16^. That is, the uncertainty in the estimation of the thermodynamic parameters is rather determined by the linear regression than the s.e.m. of the individual data points. The overall Arrhenius plot for the ribosome reaction (Fig. 4a) yields *R*^2^ = 0.84 and an r.m.s. of residuals of ~1 e.u., which equals 0.3 kcal/mol in terms of *T*Δ*S*^‡^ at 298 K. The Arrhenius plots for uncatalyzed hydrolysis of Mg^2+^•GTP^4−^ in water, via the associative and dissociative mechanisms, were based on five independent free energy profiles at each temperature (s.e.m. < 0.2 kcal/mol). For the uncatalyzed attack of OH^−^ on protonated Mg^2+^•GTP^3−^ in water ten independent free energy profiles were averaged at each temperature, with an s.e.m. < 0.16 kcal/mol.

The Mg^2+^•GTP^4–^ hydrolysis reaction mechanisms considered here were described by the EVB method^17^,^18^ with parametrization of the different models as described earlier^14^. The uncatalyzed associative and dissociative reaction pathways in solution were thus both calibrated to reproduce the experimentally derived^14^,^22^,^23^ activation free energy of 27 kcal/mol, as described in detail in ref. 14. For the stepwise mechanism involving proton transfer from the catalyctic water molecule to the γ-phosphate of GTP and subsequent attack of OH^−^, the energetics of the first step in solution was calibrated based on the p*K*_a_ difference between water and GTP, together with accurate linear free energy relationships for the (non-rate-limiting) intervening barrier^14^. This yielded activation and reaction free energies for the proton transfer step in water of 15.7 and 12.2 kcal/mol, respectively^14^. The activation free energy for hydroxide ion attack on protonated Mg^2+^•GTP^3-^ in water was estimated to be 17.5 kcal/mol at a 1 M standard state^14^, corresponding to an overall uncatalyzed reaction rate of 2 × 10^−9^ M^−1^ s^−1^ at 300 K for this mechanism.

## Additional Information

**How to cite this article**: Åqvist, J. and Kamerlin, S. C.L. Exceptionally large entropy contributions enable the high rates of GTP hydrolysis on the ribosome. *Sci. Rep.* **5**, 15817; doi: 10.1038/srep15817 (2015).

## Supplementary Material

Supplementary Information
