# Supplementary material for: Exceptionally large entropy contributions enable the high rates of GTP hydrolysis on the ribosome
Source: Sci Rep. 2015 Oct 26;5:15817. doi: 10.1038/srep15817 (PMC4620562; doi:10.1038/srep15817)
Supplement: Supplementary Information [file srep15817-s1.pdf]

Exceptionally large entropy contributions enable the high rates of GTP hydrolysis on the ribosome

Johan Åqvist & Shina C.L. Kamerlin

Dept. of Cell & Molecular Biology, Uppsala University, Biomedical Center, Box 596,

SE-751 24 Uppsala, Sweden

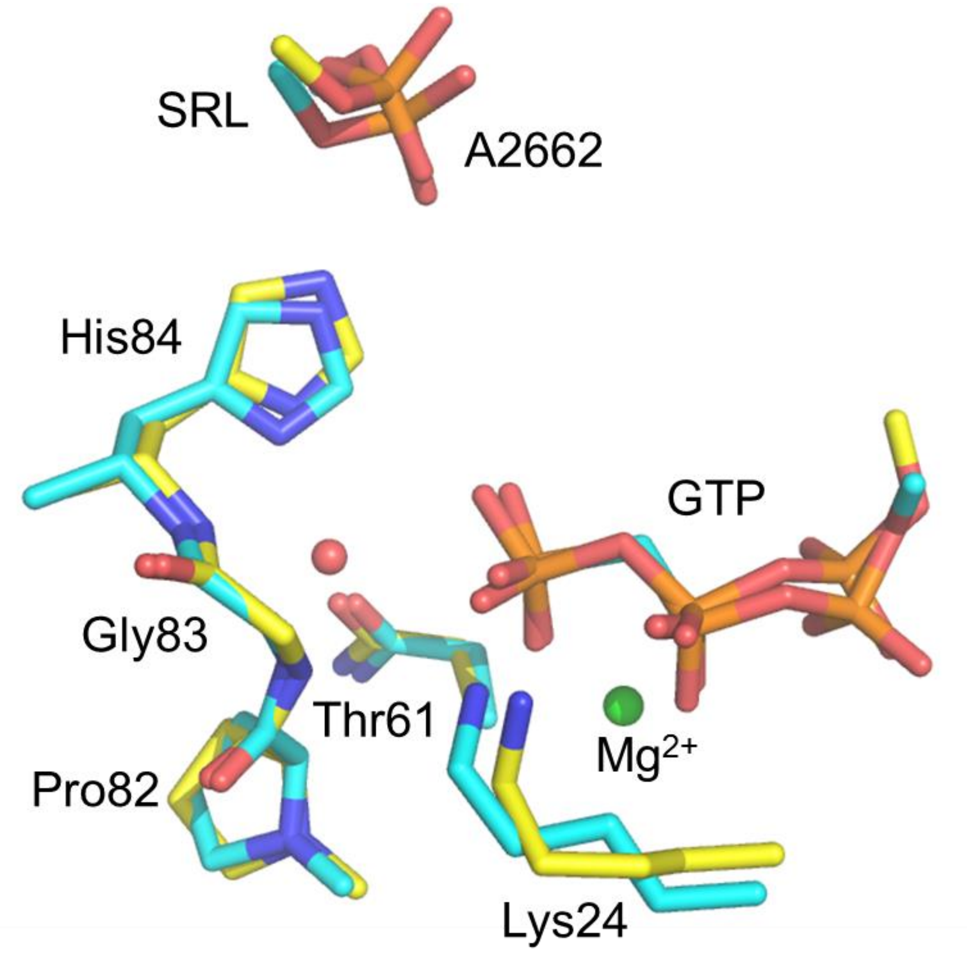

Supplementary Figure 1. Conformation of the conserved PGH motif in the translational GTPases. The best resolved recent structure[12] of the catalytic site of the translational GTPases in their active conformation (cyan carbons) is overlayed on the MD structure of the intermediate hydroxide ion state shown in Fig. 3b (yellow carbons), with the hydroxide ion and Mg^{2+} shown as red and green spheres, respectively. The specific conformation of the Pro82-Gly83 and Gly83-His84 peptide planes in the crystal structure most closely resembles that of the intermediate state in Fig. 3, which may suggest that this state has actually been captured in the experimental structure.
